# Supplementary material for: Evaluating the measurement properties of the Hindi Integrated Palliative Care Outcome Scale (IPOS) in advanced cancer patients receiving home-based palliative care in India
Source: PLOS Glob Public Health. 2026 Apr 3;6(4):e0006189. doi: 10.1371/journal.pgph.0006189 (PMC13048499; doi:10.1371/journal.pgph.0006189)
Supplement: S1 File — (DOCX) [file pgph.0006189.s001.docx]

Supplementary Online File:

**Table of Contents:**

Fig A Integrated Palliative Outcome Scale Hindi version (available to download from: www.pos-pal.org) 2

[Table A Exploratory factor analysis showing 3-factor solution with parcelled items as feasible (n=240)…………………………………………………………………………………………………………………………………………… 1](#_Toc155874416)

[Table B Paired t-test comparing mean difference Standard Deviation, 95% CI, t, df and significance of baseline and first follow-up subscale scores of patients who reported at the second assessment that they have felt the ‘same’ since the last assessment 3](#_Toc155874417)

[Table C Convergent and Divergent Validity - Spearman's Rho correlation of IPOS Hindi Subscales with EQ-5D-5L items and Health Status (n=237)](#_Toc155874418) 4

[Table D Effect sizes for subscale change scores between baseline and first-follow up assessment in persons who self-reported to have improved.](#_Toc155874419) 5

[Table E Effect sizes for subscale change scores between first and second-follow up assessment in participants who self-reported to have improved.](#_Toc155874420) 5

[Table F SRM for mean change scores from baseline to second follow-up assessment with available data 6](#_Toc155874421)

Figure B CFA Model without host-hoc modification (n=240)……………………………………………………………6

Fig A Integrated Palliative Outcome Scale Hindi version (available to download from: www.pos-pal.org)


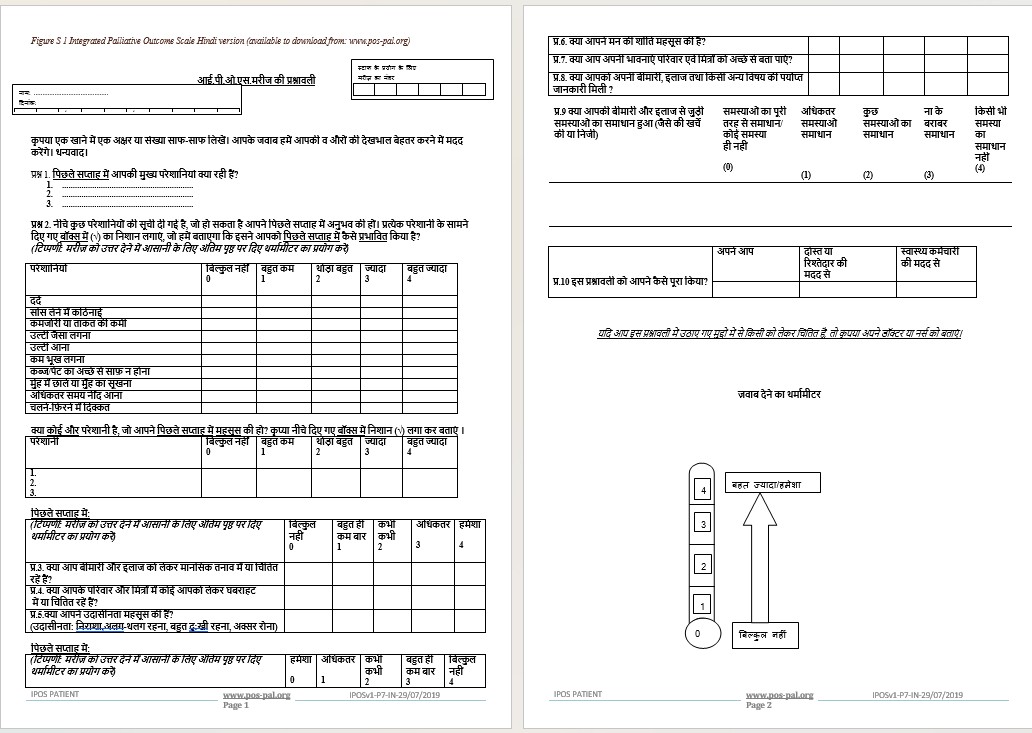


Table A Exploratory factor analysis showing 3-factor solution with parcelled items as feasible (n=240)

|  | **Physical** | **Communication &**  **Support** | **Emotional** |
| --- | --- | --- | --- |
| Poor mobility | **0.70** |  | -0.28 |
| Weakness or lack of energy | **0.61** |  | -0.40 |
| Pain | **0.53** |  | -0.27 |
| Shortness of breath | **0.47** |  | -0.25 |
| Constipation | **0.44** |  |  |
| Gastrointestinal Parcel | **0.38** |  | -0.28 |
| Poor appetite | **0.27** |  | -0.25 |
| Sore or dry mouth | **0.25** |  |  |
| Drowsiness | **0.18** |  |  |
| Practical (Personal and Financial) problems due to illness been addressed | 0.41 | **-0.25** | -0.30 |
| Sharing feelings with family or friend |  | **-0.62** |  |
| Getting information as much as wanted | 0.25 | **-0.33** | - |
| Feeling anxious or worried about illness or treatment | 0.33 | -0.28 | **-0.73** |
| Feeling depressed | 0.37 | -0.29 | **-0.72** |
| Family or friends feeling anxious or worried | 0.31 |  | **-0.58** |
| Feeling at peace | 0.34 | -0.64 | **-0.50** |
| Extraction Method: Principal Axis Factoring. | | | |
| Rotation Method: Oblimin with Kaiser Normalisation. | | | |
| Blanks represent factor loadings <0.30/-0.30 | | | |
| Loadings in bold indicate the factor the items are grouped under. | | | |

Table B Paired t-test comparing mean difference Standard Deviation, 95% CI, t, df and significance of baseline and first follow-up subscale scores of patients who reported at the second assessment that they have felt the ‘same’ since the last assessment

|  |  | **Mean Difference** | **SD** | **95% Confidence Interval of the Difference** | | **t** | **df** | **p-value (2 sided)** |
| --- | --- | --- | --- | --- | --- | --- | --- | --- |
|  | **n** |  |  | **Lower** | **Upper** |  |  |  |
| *Physical* | 37 | 0.72 | 3.91 | -0.58 | 2.02 | 1.12 | 36 | 0.27 |
| *Emotional* | 42 | 0.24 | 2.99 | -0.69 | 1.17 | 0.52 | 41 | 0.61 |
| *Communication & Support* | 41 | -0.39 | 2.26 | -1.10 | 0.32 | -1.11 | 40 | 0.27 |

Table C Convergent and Divergent Validity - Spearman's Rho correlation of IPOS Hindi Subscales with EQ-5D-5L items and Health Status (n=237)

| **IPOS Hindi Subscales** |  | | **EQ-5D-5L** | | | | | |
| --- | --- | --- | --- | --- | --- | --- | --- | --- |
| *Physical* |  | **Mobility** | | **Self-care** | **Usual Activity** | **Pain/Discomfort** | **Anxiety/**  **Depression** | **Self-rated Health (VAS)** |
|  | ***r*** | 0.53** | | 0.46** | 0.55** | 0.40** | 0.53** | -0.44** |
|  | **Sig(2-tailed)** | <.001 | | <.001 | <.001 | <.001 | <.001 | <.001 |
|  | **n** | 208 | | 208 | 208 | 208 | 208 | 205 |
| *Emotional* | ***r*** | 0.57** | | 0.50** | 0.58** | 0.44** | 0.31** | -0.41** |
|  | **Sig(2-tailed)** | <.001 | | <.001 | <.001 | <.001 | <.001 | <.001 |
|  | **n** | 213 | | 213 | 213 | 213 | 213 | 210 |
| *Communication & Support* | ***r*** | 0.29** | | 0.26** | 0.35** | 0.27** | 0.62** | -0.34** |
|  | **Sig(2-tailed)** | <.001 | | <.001 | <.001 | <.001 | <.001 | <.001 |
|  | **n** | 237 | | 237 | 237 | 237 | 237 | 234 |

**VAS** = visual analogue scale

Table D Effect sizes for subscale change scores between baseline and first-follow up assessment in persons who self-reported to have improved.

| \| **Time interval** \| **Subscale** \| **n** \| **ES** \| **Mean Change Score in Improved Group (n)** \| **SD in the group who feels the same (unchanged) since T0 assessment (n)** \| \| --- \| --- \| --- \| --- \| --- \| --- \| \| Baseline (T0) to  First follow-up (T1) Assessment \| Physical \| 35 \| -0.3 \| -1.2(35) \| 3.9(37) \| \| Emotional \| 43 \| -0.6 \| -1.8(43) \| 3.0(42) \| \| Communication  & Support \| 41 \| -0.4 \| -0.9(41) \| 2.3(41) \| |
| --- | --- | --- | --- | --- | --- | --- | --- | --- | --- | --- | --- | --- | --- | --- | --- | --- | --- | --- | --- | --- | --- | --- |

Table E Effect sizes for subscale change scores between first and second-follow up assessment in participants who self-reported to have improved.

| **Time interval** | **Subscale** | **n** | **ES** | **Mean Change Score (T2-T1) in Improved Group (n)** | **SD in the group who feels the same (unchanged) since T1 assessment (n)** |
| --- | --- | --- | --- | --- | --- |
| First follow-up (T1) to Second follow-up (T2) Assessment | Physical | 29 | -0.6 | -2.7(29) | 4.6(32) |
|  | Emotional | 32 | -0.5 | '-1.2(32) | 2.6(37) |
|  | Communication & Support | 29 | 0.0 | -0.1(29) | 2.2(35) |

Table F SRM for mean change scores from baseline to second follow-up assessment with available data

| **Subscale** | **SRM** | **Mean Change Score**  **T2-T0 in total sample (n)** | **SD of Mean Change Score T2-T0 assessment** |
| --- | --- | --- | --- |
| Physical | -0.4 | -2.3(99) | 5.6 |
| Emotional | -0.3 | -0.9(112) | 3.3 |
| Communication & Support | 0.0 | 0(131) | 2.3 |

*Fig B CFA Model without post-hoc modifications (n=240)*


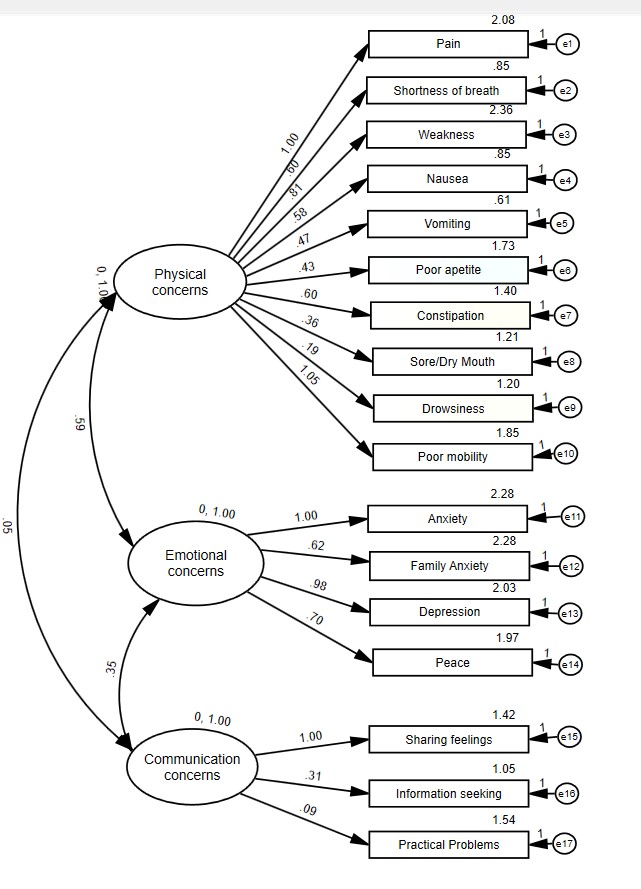


**Legend**

| Latent variables or constructs or unobserved factors | e_1_ |
| --- | --- |
| Observed or measured variables |  |
| Residual or error variance |  |
| Factor loading |  |
| covariance or correlation |  |
