# Supplementary material for: Evaluating the measurement properties of the Hindi Integrated Palliative Care Outcome Scale (IPOS) in advanced cancer patients receiving home-based palliative care in India
Source: PLOS Glob Public Health. 2026 Apr 3;6(4):e0006189. doi: 10.1371/journal.pgph.0006189 (PMC13048499; doi:10.1371/journal.pgph.0006189)
Supplement: Attachment — Submitted filename: Response to Reviewers 24.09.2025.docx [file pgph.0006189.s004.docx]

**Dated: 15.09.2025**

**To,**

**The Editor,**

**PLOS Global Public Health**

**Sub: Point-by-Point Response to the Editor and Reviewers’ Remarks**

Dear Steve Zimmerman and Reviewers,

We are pleased to resubmit our revised manuscript, titled *"Evaluating the measurement properties of the Hindi IPOS in advanced cancer patients receiving home-based palliative care in India”****,*** for consideration in your esteemed journal.

We were very moved by how thoroughly and thoughtfully the reviewers engaged with our work. Their comments reflected a deep understanding of the manuscript’s aims, and we found their suggestions both insightful and invaluable. In response to these comments, we have made substantial revisions to the manuscript, particularly in the methods, results, discussion and limitations section.

We are grateful for the opportunity to improve this manuscript and are confident that the changes have strengthened its contribution to literature on PROMs related research. We hope the revised manuscript will now meet the expectations of the PLOS GPH readership. Our Point-wise response is provided at the end of this letter, and changes are effected in the manuscript at appropriate places.

Thank you again for the opportunity to revise and resubmit, and for the thoughtful consideration of our work.

Yours Sincerely,
 Dr Mevhibe Hocaoglu
 On behalf of all co-authors

**Point-by-Point Response to Editors and Reviewers**

**Journal Requirements:**

**1. Please include a complete copy of PLOS’ questionnaire on inclusivity in global research in your revised manuscript. Our policy for research in this area aims to improve transparency in the reporting of research performed outside of researchers’ own country or community. The policy applies to researchers who have travelled to a different country to conduct research, research with Indigenous populations or their lands, and research on cultural artefacts. The questionnaire can also be requested at the journal’s discretion for any other submissions, even if these conditions are not met. Please find more information on the policy and a link to download a blank copy of the questionnaire here:** [**https://journals.plos.org/globalpublichealth/s/best-practices-in-research-reporting**](https://journals.plos.org/globalpublichealth/s/best-practices-in-research-reporting)**. Please upload a completed version of your questionnaire as Supporting Information when you resubmit your manuscript.**

**Response:** Thank you so much for bringing this important requirement to our notice. A completely filled up copy of the PLOS’ questionnaire on inclusivity in global research is now attached. Questionnaire has also been mentioned in Methods section of the manuscript.

**2. We noticed you have some minor occurrence of overlapping text with the following previous publication(s), which needs to be addressed:-** [**https://doi.org/10.1186/s12955-023-02102-4**](https://doi.org/10.1186/s12955-023-02102-4)

**In your revision ensure you cite all your sources (including your own works), and quote or rephrase any duplicated text outside the methods section. Further consideration is dependent on these concerns being addressed.**

**Response:** The overlapping text is now appropriately paraphrased and referenced.

**Reviewers’ comments**

**Reviewer #1: The study addresses a critical gap by validating a culturally adapted tool for Hindi-speaking patients.

Major Limitations and Suggestions**

**1. The internal consistency (Cronbach’s alpha = 0.35) is well below acceptable thresholds. Item-total correlations show weak discrimination, particularly for "Practical Problems" (0.14). Consider revising or expanding this domain for future iterations.**

**Response:** Thank you. It is a very important point. We have worked on this point and added the following paragraph in discussion section along with appropriate reference.

“The internal consistency of the third subscale was relatively low in our sample (Cronbach’s α = 0.35). Notably, the original validation study also reported modest reliability for this subscale (α = 0.58), suggesting that this dimension of the scale may have inherent limitations in internal consistency. Possible explanations include the small number of items, the heterogeneous nature of the construct, and contextual differences in how participants interpreted the items. While this indicates that findings related to this subscale should be interpreted with caution, the content remains theoretically important. Future work should consider revising or expanding this subscale and applying alternative reliability assessments e.g., McDonald’s omega, test–retest to better capture the construct in diverse settings.”

**2. Twelve items show significant floor effects (≥15%), limiting sensitivity to change in less symptomatic patients. Authors should consider item response theory-based recalibration or alternate scoring techniques.**
Response: Thank you for this very important observation. Given the observed floor effects, robust calibration will likely require larger, population-based samples to improve item coverage at the lower end of the trait and stabilize parameter estimates. Our current study sample was too small to permit such calibration; however, we plan to explore this in future work through population-based implementation.  We have now added in the discussion section the following sentence along with appropriate reference.

“The observed floor effects suggest that robust calibration will require larger, population-based samples. Our current study was underpowered for this, but we plan to address it in future population-based work”.

**3. Pairwise deletion risks bias, and exclusion of subscale scores with single missing items limits clinical applicability. The authors might explore multiple imputation or prorated scoring options.**

**Response:** Thank you very much for raising very important point. This is a good point. The measure was administered by the community team, and the number of missing items was very small. We have added the following:

“If missingness were to be high in future studies, we recommend considering appropriate imputation approaches; however, during initial validation studies, most authors caution against imputation, as it may distort psychometric properties”.

We have also quoted the reference to support our justification.

4**. The three-factor structure required parceling of GI symptoms to achieve acceptable CFA fit indices. While justifiable, this alters the original IPOS construct and weakens cross-cultural comparability. Explicit justification and sensitivity analysis are encouraged**.

**Response**: We appreciate this important comment. The decision to parcel the GI symptom items was based on both conceptual relevance (these items represent higher-prevalence and clinically salient symptoms in our population) and statistical necessity to achieve acceptable CFA fit. We note that similar strategies have been employed in prior IPOS psychometric work which lends precedent to this approach. We agree that parceling alters the original construct and may weaken cross-cultural comparability; therefore, we have added explicit justification in the manuscript and conducted a sensitivity analysis to assess the robustness of our findings.
We have added this paragraph to the discussion section as a limitation:

“The three-factor model required parcelling of GI items to achieve acceptable fit. Without parcelling, fit indices were poor, indicating that this subdomain was unstable in our sample. While this justifies the analytic choice as similar techniques were used in other IPOS studies too, it alters the original IPOS construct and should be interpreted with caution. Future studies with larger, more diverse samples should confirm the factor structure without parceling”.

**5. The COVID-19-related restriction to Delhi NCR reduces generalizability to other Hindi-speaking regions (e.g., UP, Bihar). This should be more clearly stated in the limitations.**

**Response:**  Thank you for this important observation. We agree that restricting recruitment to the Delhi NCR region during COVID-19 limits the generalizability of our findings to other Hindi-speaking populations, such as those in Uttar Pradesh and Bihar. We have now made this explicit in the limitations section of the manuscript.

“Due to COVID-19 restrictions, data collection was restricted to Delhi NCR. Patients could not be recruited from other nearby states such as Uttar Pradesh, Haryana and Punjab in India, therefore this may affect the generalisability of our findings to Hindi Speaking populations in other regions”.

**6. The sample includes only cancer patients. Validation for other chronic illnesses (e.g., organ failure, neurodegenerative diseases) is necessary to extend applicability of Hindi IPOS. In case that is not possible, the same should be explicitly mentioned.**

**Response**: Thank you so much for your remarks. We appreciate this valuable point and completely agree that validation of IPOS in other chronic illnesses e.g., organ failure, neurodegenerative diseases) is required. This study focuses only on cancer patients, however future studies are required to assess IPOS validation for other chronic conditions. We have explicitly mentioned this in manuscript in future research section following your suggestions.
We have added the following lines in limitation section:

“Our sample comprised only patients with cancer, and therefore the applicability of the Hindi IPOS to other chronic illnesses (such as organ failure or neurodegenerative conditions) remains to be established. Future validation work in these populations is needed before wider use can be recommended.”

**7. Word cloud visualization offers limited insight into rich psychosocial concerns expressed in responses. A thematic analysis with illustrative quotes could enhance content validity and support item refinement.**

**Response**: We thank the reviewer for this thoughtful suggestion. We agree that word cloud visualization has limited interpretive depth. The qualitative data in our study were limited to short open-text responses, which constrained the depth of analysis possible. For this reason, we opted for word cloud visualization as a descriptive approach. Due to resource and scope constraints, we were not able to conduct a full thematic analysis, but we have clarified this limitation in the manuscript and acknowledged that future qualitative work, including thematic analysis with illustrative quotes, could enhance content validity and guide item refinement. We agree that future studies using richer qualitative methods (e.g., interviews or focus groups) could enable thematic analysis with illustrative quotes to strengthen content validity and inform item refinement. We have added this clarification to the manuscript.

“The qualitative component in our study was based on brief open-text comments, which limited the depth of qualitative analysis. While we presented word cloud visualizations for descriptive purposes, future work using more in-depth qualitative methods (e.g., interviews, focus groups) will be important to generate richer thematic insights and support item refinement.”

**8. Low engagement with "information" and "sharing feelings" items may reflect cultural norms or literacy barriers, or lack of trust between interviewer and responder. These require deeper investigation in future studies.**

**Response:** We appreciate this observation. We agree that low engagement with these items may reflect cultural or contextual factors rather than irrelevance. We have now noted this in the discussion and recommended further investigation of cultural norms, literacy, and patient–interviewer dynamics in future studies. We have added the following line in discussion section:

“Low engagement with the ‘information’ and ‘sharing feelings’ items may reflect cultural norms, literacy barriers, or trust dynamics between participants and interviewers rather than lack of relevance. These factors warrant deeper investigation in future research.”

**9. Emotional and Communication subscales showed weak responsiveness and no discriminative validity by disease stage. This questions their use in longitudinal or intervention-based studies.**
**Response:** We agree with the reviewer’s interpretation and have now acknowledged this explicitly in the manuscript. To the limitations section we are adding the following sentence:

“The Emotional and Communication subscales showed weak responsiveness and did not discriminate by disease stage, which raises concerns about their utility in longitudinal or intervention-based studies.”

**10. Minimally Important Difference (MID) estimates are based on small subgroups (n=20–43), constraining precision. Bootstrapped confidence intervals or anchor-based validation in larger samples may be required.**

**Response**: We thank the reviewer for highlighting this limitation. We have clarified in the manuscript that our MID estimates are preliminary and constrained by small subgroup sizes, and we recommend bootstrapped CIs and anchor-based validation in larger samples for future work. We have added in limitations:

“Our estimates of Minimally Important Difference (MID) were based on small subgroups (n=20–43), which constrains precision. These findings should be considered preliminary, and future studies with larger samples should confirm them using bootstrapped confidence intervals and anchor-based validation.”

**11. Minor Issues**
Typographical inconsistencies (e.g., “preset” study instead of “present”) should be corrected.
**Response:** Thank you so much. Typographical errors are now corrected.

12.**Figures (CFA model, word cloud) lack clear legends and numeric labels; revise for readability.**

**Response:** Figures are now revised for clear readability.

**13. Data availability constraints could be elaborated to clarify ethical restrictions vs. operational barriers.**

**Response:** Thank you so much for raising this important point. We have now inserted the following statement to the data sharing section at the end of the manuscript:

“Data cannot be shared publicly due to ethical restrictions related to patient confidentiality and operational barriers in accessing institutional records. De-identified data may be available upon reasonable request to the corresponding author, subject to ethics committee approval.”

Thank you so much.
