# Supplementary material for: Evaluating the measurement properties of the Hindi Integrated Palliative Care Outcome Scale (IPOS) in advanced cancer patients receiving home-based palliative care in India
Source: PLOS Glob Public Health. 2026 Apr 3;6(4):e0006189. doi: 10.1371/journal.pgph.0006189 (PMC13048499; doi:10.1371/journal.pgph.0006189)
Supplement: Attachment — Submitted filename: Review Comment.docx [file pgph.0006189.s005.docx]

**Title**-  Evaluating the measurement properties of the Hindi **IPOS** in advanced cancer patients receiving home-based palliative care in India-  Abbreviations should be defined when mentioned for the first time to ensure clarity for readers who may not be familiar with the term.

**Abstract**

Methods-

1. The manuscript states that *“This validation study was conducted with 240 adults living with cancer and receiving home-based palliative care in the Delhi National Capital Region of India.”* Given that Delhi/NCR is a highly populated region and the national capital, the sample size of 240 patients appears relatively small compared to the total home-based palliative care population. It would strengthen the methodology section if the authors clarify **from which specific centre(s) / home-based palliative care programme(s)** these patients were recruited.

2.  EQ-5D-5L - Abbreviations should be defined when mentioned for the first time to ensure clarity for readers who may not be familiar with the term.

Results

  The statement regarding construct and known-groups validity being “supported by significant associations with EQ-5D-5L and cancer stage” remains incomplete without the presentation of statistical evidence. Please include the corresponding p-values in the results section to substantiate this claim.

Please mention the secondary objective too

**Methodology**

  1. Please clarify whether all participant information was collected telephonically. If so, this should be stated clearly in the methodology section of the abstract, as the mode of data collection is an essential methodological detail.

2. Clarity regarding patient recruitment. Please indicate the number of eligible patients during the study duration, describe how they were approached, and report the final number enrolled. This information is essential for readers to evaluate the sampling process and overall robustness of the study design.

3. Please mention the inclusion and exclusion criteria of the participants

**Results**

 1. The term “senior secondary (14 years of education)” is ambiguous without specifying the corresponding school standard. Please clearly state which class/grade this refers to (e.g., Class 12)

 2.  Socioeconomic status is referenced in the results, but the methodology does not describe how it was defined or assessed.

3. The presence of Stage I and II patients in a home-based palliative care cohort seems inconsistent with standard palliative care referral patterns, which predominantly include advanced-stage cases. Please clarify the staging assessment method and the referral criteria that resulted in the inclusion of early-stage patients.

**Discussion**

1. The authors justify the use of closed-ended questions by stating that such formats facilitate comparisons across groups. Since this is a single-group study, this rationale does not align with the methodology.
