# Supplementary material for: Evaluating the measurement properties of the Hindi Integrated Palliative Care Outcome Scale (IPOS) in advanced cancer patients receiving home-based palliative care in India
Source: PLOS Glob Public Health. 2026 Apr 3;6(4):e0006189. doi: 10.1371/journal.pgph.0006189 (PMC13048499; doi:10.1371/journal.pgph.0006189)
Supplement: Attachment — Submitted filename: Response to Reviewers Comments and Feedback 07.01.2026.docx [file pgph.0006189.s006.docx]

**Dated:** 19 December 2025

**To**
Dr Alejandro Torrado Pacheco
Editor
*PLOS Global Public Health*

**Subject:** Point-by-Point Response to the Editor’s and Reviewers’ Comments

Dear Dr Torrado Pacheco,

We are pleased to resubmit our revised manuscript entitled **“Evaluating the measurement properties of the Hindi Integrated Palliative Care Outcome Scale (IPOS) in advanced cancer patients receiving home-based palliative care in India”** for consideration in *PLOS Global Public Health*.

We sincerely thank the Editor and Reviewers for their thoughtful, constructive, and detailed feedback. We are grateful for the opportunity to revise the manuscript and believe that the changes made have substantially strengthened the clarity, methodological transparency, and overall contribution of the study to the literature on patient-reported outcome measures (PROMs) in palliative care and global public health. We hope the revised manuscript now meets the expectations of the journal’s readership.

A detailed, point-by-point response to all comments is provided below. All revisions have been incorporated at appropriate locations in the manuscript.

Thank you again for your time and consideration.

Yours sincerely,
**Dr Mevhibe Hocaoglu**
On behalf of all co-authors

**Point-by-Point Response to the Editor and Reviewers**

**Journal Requirements**

**Comment:**
If reviewer comments include recommendations to cite specific previously published works, authors should review and evaluate these publications.

**Response:**
Thank you. No such recommendations were made by the reviewers.

**Reviewer #1**

**1. Communication & Support Subscale**

**Comment:**
Internal consistency remains low (α = 0.35). Future iterations should consider item expansion or alternative reliability metrics.

**Response:**
Thank you for this observation. This point has now been incorporated into the Discussion. The revised text reads:

“Internal consistency for this subscale was low (Cronbach’s α = 0.35). Future work may benefit from item expansion and the use of alternative reliability indices, such as McDonald’s omega or test–retest reliability, to better characterise the construct.”

**2. Floor Effects**

**Comment:**
Twelve items show ≥15% floor effects. Consider item response theory (IRT) or recalibration in future studies.

**Response:**
Thank you for this valuable suggestion. This has now been addressed in the Discussion, which reads:

“Twelve items demonstrated floor effects (≥15%), which may have limited sensitivity at the lower end of the scale. Similar item-level floor effects have been reported in previous IPOS studies. Future work may consider item response theory–based approaches or recalibration to improve measurement performance.”

**3. Generalizability**

**Comment:**
Sample restricted to Delhi NCR due to COVID-19. Future validation in other Hindi-speaking regions and non-cancer populations is essential.

**Response:**
This limitation has been explicitly acknowledged in the Discussion.

**4. Qualitative Depth**

**Comment:**
Word cloud visualization is informative but limited. Future studies should incorporate thematic analysis with illustrative quotes.

**Response:**
Thank you for this helpful comment. This has now been incorporated into the Discussion:

“Word cloud visualisation provides a useful descriptive overview but offers limited analytic depth. Future studies may benefit from thematic analysis with illustrative quotes, potentially triangulated with qualitative interviews, to strengthen interpretive depth and content validity.”

**5. Figures and Formatting**

**Comment:**
Ensure all figures have clear legends and numeric labels; final proofread recommended.

**Response:**
Figures have been revised to include clear legends and numeric labels, and the manuscript has been thoroughly proofread.

**6. Data Sharing Statement**

**Comment:**
Ethical and operational constraints are articulated; consider including a data access protocol.

**Response:**
Thank you for raising this important point. A data access protocol has now been included. The revised Data Availability statement reads:

*The minimal data set underlying the results presented in this manuscript—including values underlying summary statistics, measures of variation, and data used to generate figures and tables—may be made available upon reasonable request. Due to ethical and confidentiality restrictions protecting participant privacy, the full dataset cannot be made publicly available. The Institutional Ethics Committee – CanSupport and the collaborating academic institution, Dr Bhim Rao Ambedkar College, University of Delhi, have imposed restrictions on public data sharing, as certain demographic variables and free-text responses may allow participant re-identification even after de-identification. Researchers seeking access to the minimal de-identified dataset should submit a formal request to the Palliative Outcome Scale (POS) Team (palliativecare@pos-pal.org), including a brief description of the proposed analyses and data protection arrangements. Requests will be reviewed in accordance with institutional governance and ethical approval requirements, and approved access will be subject to a data use agreement.*

**Reviewer #2**

**Comment:**
Positive overall assessment.

**Response:**
We thank the reviewer for their encouraging and supportive comments.

**Reviewer #3**

**Title**

**Comment:**
Define abbreviations at first mention.

**Response:**
The title has been revised to define IPOS in full.

**Abstract – Methods**

**1. Recruitment Source**

**Comment:**
Clarify recruitment centre(s).

**Response:**
We have specified in both the Abstract and Methods that participants were recruited from a single charitable home-based palliative care organisation (CanSupport).

**2. EQ-5D-5L Abbreviation**

**Response:**
The full name, *EuroQol 5-Dimension 5-Level questionnaire (EQ-5D-5L)*, is now defined at first mention.

**Results**

**3. Statistical Evidence**

**Comment:**
Include p-values to support claims of construct and known-groups validity.

**Response:**
Corresponding p-values (p < 0.001) have been added to the Abstract and Results sections.

**4. Secondary Objective**

**Comment:**
Please mention the secondary objective.

**Response:**
This study was designed with a single primary objective: to evaluate the measurement properties of the Hindi IPOS. No secondary objectives were pre-specified. This has now been stated explicitly in the Abstract and Introduction.

**Methodology**

**1. Mode of Data Collection**

**Comment:**
Clarify whether all participant information was collected telephonically.

**Response:**
Participant recruitment and baseline and follow-up assessments were conducted in person by home-based palliative care teams. Only a small subset of participants (n = 10) completed the third follow-up assessment via video call due to COVID-19–related constraints. No data were collected telephonically. This has been clarified in the Abstract and Methods.

**2. Patient Recruitment**

**Comment:**
Clarify eligible population, recruitment process, and final enrolment.

**Response:**
The following text has been added to the Methods section:

“During the recruitment period (February–March 2021), approximately 400 patients received services from the home-based palliative care programme. All eligible patients were approached during routine visits. Of these, 240 consented and were enrolled in the study.”

**3. Inclusion and Exclusion Criteria**

**Response:**
Inclusion and exclusion criteria have now been explicitly stated in the Methods section.

**Results**

**1. Education Category**

**Comment:**
Clarify “senior secondary.”

**Response:**
The corresponding class (Class 12) has been specified in the table and footnote.

**2. Socioeconomic Status**

**Comment:**
Clarify how socioeconomic status was assessed.

**Response:**
We have clarified that socioeconomic variables were self-reported, while clinical data were obtained from medical records.

**3. Cancer Staging**

**Comment:**
Clarify inclusion of early-stage patients.

**Response:**
Cancer stage was recorded from hospital documentation at referral. The home-based palliative care service does not independently stage disease, and referrals are needs-based rather than stage-based. This has been clarified in the Methods.

**Discussion**

**Closed-Ended Items**

**Comment:**
Justification for closed-ended items was inconsistent with a single-group study.

**Response:**
This has been revised to clarify that IPOS is a standardized, non-individualised measure with fixed scored items, and that analyses followed COSMIN methodology focusing on the scored components of the instrument.

**Figure Resubmissions**

**Response:**
Figures were processed using the PLOS NAAS tool and meet all technical requirements. Updated figures have been uploaded with the revised manuscript.
